# Brno University of Technology Smartphone PPG Database (BUT PPG): Annotated Dataset for PPG Quality Assessment and Heart Rate Estimation

**DOI:** 10.1155/2021/3453007

**Published:** 2021-09-06

**Authors:** Andrea Nemcova, Enikö Vargova, Radovan Smisek, Lucie Marsanova, Lukas Smital, Martin Vitek

**Affiliations:** ^1^Department of Biomedical Engineering, Faculty of Electrical Engineering and Communication, Brno University of Technology, Technicka 12, Brno 616 00, Czech Republic; ^2^Institute of Scientific Instruments, The Czech Academy of Sciences, Kralovopolska 147, Brno 612 64, Czech Republic

## Abstract

To the best of our knowledge, there is no annotated database of PPG signals recorded by smartphone publicly available. This article introduces Brno University of Technology Smartphone PPG Database (BUT PPG) which is an original database created by the cardiology team at the Department of Biomedical Engineering, Brno University of Technology, for the purpose of evaluating photoplethysmographic (PPG) signal quality and estimation of heart rate (HR). The data comprises 48 10-second recordings of PPGs and associated electrocardiographic (ECG) signals used for determination of reference HR. The data were collected from 12 subjects (6 female, 6 male) aged between 21 and 61. PPG data were collected by smartphone Xiaomi Mi9 with sampling frequency of 30 Hz. Reference ECG signals were recorded using a mobile ECG recorder (Bittium Faros 360) with a sampling frequency of 1,000 Hz. Each PPG signal includes annotation of quality created manually by biomedical experts and reference HR. PPG signal quality is indicated binary: 1 indicates good quality for HR estimation, 0 indicates signals where HR cannot be detected reliably, and thus, these signals are unsuitable for further analysis. As the only available database containing PPG signals recorded by smartphone, BUT PPG is a unique tool for the development of smart, user-friendly, cheap, on-the-spot, self-home-monitoring of heart rate with the potential of widespread using.

## 1. Introduction

PPG signals are commonly used nowadays because the optical sensors can be integrated in almost any device (smartphone, smartwatch, smartring,…). The recording of PPG is cheap, simple, noninvasive, and comfortable, and it can be done on-the-spot. In case of using any smartphone, no additional device is necessary. The PPG signal is commonly used for heart rate estimation.

Evaluation of PPG signal quality and estimation of HR from PPG signal have become a popular research topic [1–6], driven in part by the increased use of smartphones and wearables for health monitoring. A majority of HR estimation smartphone applications are neither tested, nor certified and moreover are not verified by medical experts (except, e.g., Preventicus Heartbeats [4]). Commercial smart watches such as Garmin [7] and Polar [8] also report problems with accuracy of HR estimation (caused, e.g., by physical activities, worsen blood flow in periphery, improperly weared watches, tattoo, measuring in water). Thus, HR estimation from mobile devices and wearables is still a challenging problem. It is logical for health monitoring algorithms or applications to carry out signal quality estimation before HR estimation. This approach may lead to greater robustness and reliability. For example, if a poor PPG signal quality is detected by a monitoring device, the signal can be discarded (preventing distorted and unreliable results) and a new measurement can be requested.

There exist some PPG database. However, to the best of our knowledge, there is no annotated database of PPG signals recorded by smartphone publicly available. The BUT PPG database is unique in this sense. There are some benefits of PPG recording by smartphone comparing to the smartwatch/smartrings: it is widely available and cheap; the quality of PPG is satisfactory; application for PPG-based HR estimation and evaluation can be simply integrated. As the data acquisition as well as processing can be done in one device, there is no problem with (wireless) data transmission. BUT PPG database was created for the purpose of developing and evaluating algorithms designed to assess the quality of PPG records and algorithms designed for estimation of HR from PPG.

**Figure 1:**
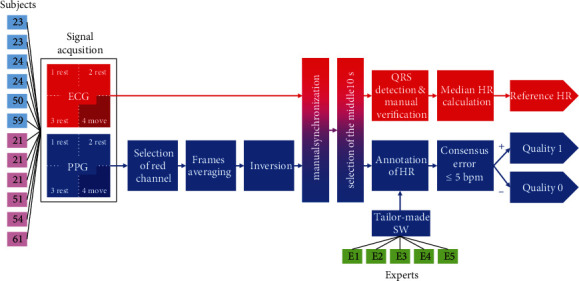
Schematic overview of the study. The diagram shows the process of creating the BUT PPG database.

Study design overview is shown in Figure 1. It starts with the subjects (on the left side)—volunteers on which the signals were acquired. Altogether, 6 men (light blue color) and 6 women (pink color) of various ages (number in each block) were measured. Two signals were acquired simultaneously—ECG signal (red color in Figure 1) using mobile ECG recorder and PPG signal (blue color in Figure 1) in a form of video using smartphone. Each subject was measured four times—three times in rest and once in move. The measured video was processed in three steps to obtain the PPG signal. The red video channel was selected, and each video frame was averaged to obtain a single value. The calculated PPG signal was inverted. Then, the ECG and PPG signals were manually synchronized, and the middle 10 seconds were selected to eliminate transition artifacts. The PPG quality and HR annotations were calculated. The HR annotations were created using the ECG signal. The QRS complexes were automatically detected and manually verified. Then, the median HR was calculated, and the output is the reference HR. The annotations of PPG signal quality were created by 5 experts using tailor-made annotating software. The experts estimated the HR from the PPG signals. Thereafter, the consensus of experts was made, and each signal was classified into good quality class (1) or poor quality class (0). All parts of the study are described below.

## 2. Materials and Methods

### 2.1. Signal Acquisition

All human studies were approved by the Institutional Review Board of DBME Faculty of Electrical Engineering and Communication, Brno University of Technology, on July 27, 2018 (IRB Protocol EC:EK:05b/2018). Informed written consent was obtained from all subjects prior to the studies.

We recorded 48 10-second PPG signals using the Xiaomi Mi9 smartphone. The measured subject attached his/her index finger to the rear side of the smartphone to cover the camera and LED light. The light was turned on, and a 30-second video of the finger was recorded. Concurrently, the single-lead ECG signal was recorded as a reference for HR. The Bittium Faros 360 device was used for this purpose. The electrodes were attached to the chest according to the device manual. The database is balanced in terms of gender (6 female records and 6 male records) and age (21 to 61 years, mean 36 years, median 24 years). Each subject was measured four times. The first three measurements were in rest while the subject was sitting. During the fourth measurement, the subject was walking and/or moving with the finger on the lens.

**Figure 2:**
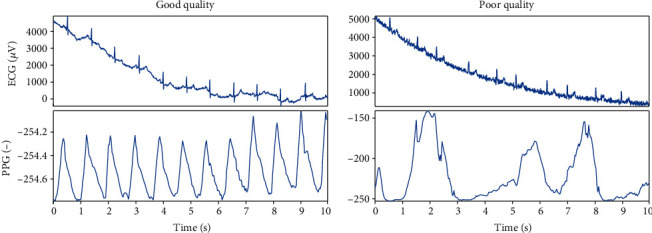
Examples of good (signal no. 6) and poor (signal no. 28) quality PPG signals. In both cases, the PPG signals are shown together with the reference ECG signals.

For the database, only the middle 10 seconds of recordings were included to eliminate signal fluctuations. From the video with resolution of 720 × 1280 px, only the red color channel [9] was considered, and the PPG signal was created using averaging of each frame. Finally, the PPG signal was inverted. The sampling frequency was 30 Hz (frames per second). The sampling frequency of the ECG signal was 1,000 Hz. PPG and ECG signals were synchronized manually. The example of PPG signals of good and poor quality together with the reference ECG signals are shown in Figure 2.

### 2.2. Annotating

In the BUT PPG database, the HR and the quality of the signals are annotated. The annotations are provided for the whole 10-second signals (one signal, one number of HR, and one quality label).

The reference HR was calculated from the ECG signals which are considered as the gold standard. The QRS complexes were found using a robust QRS detector based on a combination of three independent methods [10] and manually verified. Then, the median HR was calculated (one number for the whole 10 second signal), and this number was considered as the reference HR for the PPG signal.

PPG signals were annotated in terms of their quality. The database contains binary signal-quality labels:

- 1: indicates good quality for HR estimation
- 0: indicates poor-quality signals where the HR cannot be detected reliably and thus unsuitable for further analysis

Quality annotations are based on HR estimation using PPG signals and comparison with reference (ECG) value of HR. Each PPG signal was annotated by 5 experts in terms of HR. Annotators were provided with tailor-made software (described below) for the purpose of annotating. Annotators also had the opportunity to decline to estimate the HR when the estimate would not be reliable. Annotators saw only the PPG signals (ECG signals were not provided for the objectivity of the annotations). Moreover, the PPG signals appeared in a random manner. The annotators did not know whether the signal was recorded in rest conditions or under the artifact scenario. Signals from one subject were not annotated consecutively (this could influence the annotators because HR is similar for resting PPGs of one subject).

Once all the experts completed the HR estimations, we attempted to reach consensus for quality annotations. Only the “good” annotations were considered, meaning those HR values that differed from the reference HR by less than or equal to 5 bpm. The maximal error value of ±5 bpm is based on the international standard IEC 60601-2-27 [11]. The norm enables accuracy of ±10% or ±5 bpm (higher of these values) [11]. In the BUT PPG database, all the HR values are higher than 60 bpm; thus, the value of 5 bpm selected by us is even more strict than the norm. When at least three annotators provided good annotations, the signal was labelled as 1 (“good quality”). When this condition was not met, the signal was labelled as 0 (“poor quality”).

### 2.3. Annotating Software

**Figure 3:**
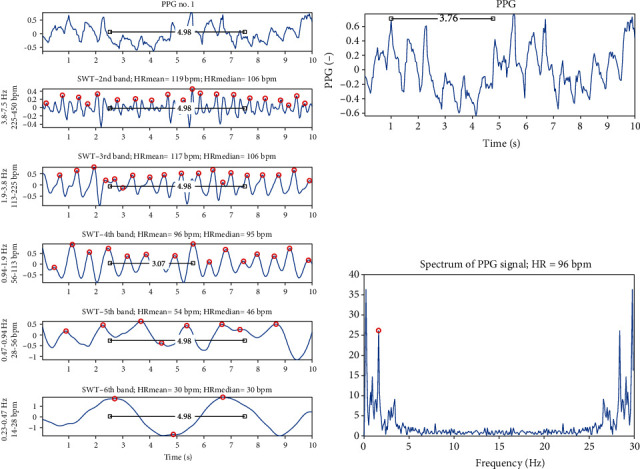
The main window of the tailor-made annotating software. (a) The original PPG signal. The five graphs on (b) show the selected frequency bands of the signal decomposed by the SWT. (c) The original PPG signal plotted especially for the manual measurement of the distances between peaks. (d) The spectrum of the signal. Red circles represent automatically found peaks of the signals. Black lines with numbers in the middle are the rulers for the manual measurement of distances between peaks within the signals.

The main window of the tailor-made annotating software is shown in Figure 3. The software includes the picture of the original PPG signal (left and right upper graphs), decomposition of the PPG signal using stationary wavelet transform (SWT) (five graphs on the left side), mean and median HR estimated from each frequency band, spectrum created using fast Fourier transform (right lower graph), and HR estimated from spectrum.

In Figure 3, the 2nd to the 6th frequency bands after SWT are shown. In each of them, the peaks are automatically found and marked by the red circles. Automatic detection of peaks was provided by finding local maxima using minimum peak distance rule (set to 8) and minimum peak prominence rule (set to 0.01). Using the distance between marked peaks, the mean and median HR are calculated and shown in the title of each graph. All the graphs (except for spectrum) are equipped with a ruler (black line with the number in the middle) for the manual measurement of the distances between peaks within the signals. From the spectrum, the HR was determined automatically as the maximum in the frequency range of 0.5-4 Hz, which corresponds with HR of 30-240 bpm, and it is shown in the title of the spectrum.

## 3. Results

### 3.1. Data Records

The data are publicly available on PhysioNet [12, 13].

Each record contains PPG signal and one-lead ECG signal recorded with a sampling frequency of 30 Hz and 1,000 Hz, respectively. All signals are provided in the WaveForm Database (WFDB) format. The names (IDs) of the recordings are six-digit numbers where the first three numbers are unique subject identifiers and the next three numbers indicate the measurement number of this subject. The PPG and ECG signals are in two separate files: ∗*_PPG.dat*, ∗*_PPG.hea*, and ∗*_ECG.dat*, ∗*_ECG.hea*, respectively.

The annotations *QUALITY-HR-ANN.csv* are recorded in a CSV file with three columns. The first column contains signal IDs. The second column contains a binary quality indicator (1: “good quality”; 0: “poor quality”). The third column contains the reference HR.

The raw HR estimations from each annotator are available in *raw-HR-annotations.csv*. Patient demographics (gender, age, and weight) and motion information are provided in *subject-info.csv*.

### 3.2. Technical Validation

In Table 1, there are shown median absolute errors between experts' PPG HR estimations and ECG HR reference. Two experts have median error of 1.5 bpm, and three of them have 2 bpm. Moreover, Table 1 shows the number of signals with HR error equal or lower than 5 bpm (based on the international standard IEC 60601-2-27 [11]) for each expert.

In Table 2, it is shown how annotators' results match with the reference. Altogether, 31 signals were classified as quality 1 (“good quality”) according to all annotators (the error of annotators' HR estimation was lower than or equal to 5 bpm in comparison with the reference). Three signals were classified as quality 1 (“good quality”), when 4 annotators' HR estimations match with the reference. Three-annotator match was reached for only one signal no. 12 (the lowest certainty). In two signals, only 2 annotators match with reference; thus, the signal was classified as quality 0 (“poor quality”) with the lowest certainty. In three signals, the HR was correctly estimated by one expert; thus, the signals belong to quality 0 (“poor quality”) class. In 8 signals, none of the experts estimated HR correctly; thus, these signals belong unambiguously to quality 0 (“poor quality”). From this table, it is evident that in case of 39 of 48 signals, the signals were classified into one of the two groups according to all of the experts. In 6 signals, only one expert was not in accordance with others. Only three signals were classified into the particular group according to three experts.

Both tables show that there is only a small variance between annotators. Tables 1 and 2 prove that the annotations of PPG signal quality are reliable.

## 4. Discussion

The reference HR annotations were made by ECG expert with the use of an advanced QRS complex detection algorithm [10]. All the ECG signals were manually checked whether the median HR can be determined from them. Thus, the reference HR annotations can be considered as reliable.

All five ECG experts who annotated the PPG signals are biomedical engineers with master's or doctoral degrees. All of them have at least 10-year experience with processing and analysis of biomedical signals. Two of them have a 7-year practice from a cardiology clinic and have experience with annotating biomedical signals [14, 15].

According to our results, 10 s PPG signal is appropriate to precisely estimate the HR (analogous to HR estimation during standard clinical ECG procedure). Another advantage of 10 s signals is that there is minimal risk of data loss during measurement and presence of artifacts (when they were not evoked) unlike long-time measurements. Short-time measurements are also comfortable for the measured subjects and do not limit them. Short-time measurement has low demands on energy, battery capacity, and memory. Moreover, BUT PPG database is quite diverse—the data were measured both in rest and in move, subjects aged 21-63 participated. It includes HR annotations and annotations of PPG signal quality.

The described results and annotations contribute to credibility of smartphone use for medical purposes—home self-monitoring of HR (by anyone—children, adults, seniors, athletes,…).

### 4.1. Limitation of the Study

BUT PPG database includes 48 10 s signals, which can be insufficient, e.g., PPG signal analysis based on artificial intelligence. This paper includes enough methodological information to enable future extensions of the database by our team (which is planned) or even by other teams.

## 5. Conclusion

As the only available database containing PPG signals recorded by smartphone, BUT PPG is a unique tool for the development of smart, user-friendly, cheap, on-the-spot, self-home-monitoring of HR with the potential of widespread using. A benefit is that it includes PPG quality annotations which can enhance the robustness and reliability of HR estimation algorithms. BUT PPG with PPG quality annotations can be used also separately for developing and testing quality estimation algorithms. One of the unique features of the database is that the quality annotation of each PPG signal is based on 5 experts and their consensus, meeting international standard guidelines, and moreover meeting the more strict variant.

This database has a great potential to contribute in the swiftly developing area of well-being, telemedicine, and self-monitoring in home environment by the omnipresent cell phone.

## Figures and Tables

**Table 1:** Successfulness of PPG expert annotators.

	E1	E2	E3	E4	E5
Median error (bpm)	1.5	1.5	2	2	2
No. of errors ≤5 bpm	36	36	34	35	36

**Table 2:** Match rate of experts with the reference ECG HR.

	Match of experts with reference ECG HR					
	5×	4×	3×	2×	1×	0×
No. of signals	31	3	1	2	3	8

## Data Availability

The data are publicly available on PhysioNet: https://physionet.org/content/butppg/1.0.0/.
